# Venetoclax in relapsed/refractory blastic plasmacytoid dendritic cell neoplasm with central nervous system involvement: a case report and review of the literature

**DOI:** 10.1186/s13256-021-02939-7

**Published:** 2021-06-26

**Authors:** Nil Albiol, Silvana Novelli, Anna Mozos, Marta Pratcorona, Rodrigo Martino, Jorge Sierra

**Affiliations:** 1 Haematology Department. Hospital de la Santa Creu i Sant Pau, Mas Casanovas 90, Barcelona, Spain; 2 Josep Carreras Leukaemia Research Institute (Hospital Sant Pau Campus), Barcelona, Spain; 3 Pathology Department. Hospital de la Santa Creu i Sant Pau, Barcelona, Spain

**Keywords:** Blastic plasmacytoid dendritic cell neoplasm, Bcl-2, Venetoclax, BPDCN

## Abstract

**Background:**

We describe a patient with blastic plasmacytoid dendritic cell neoplasm with central nervous system involvement and the outcome of venetoclax use in this setting.

**Case presentation:**

A 54-year-old Caucasian male was referred to the Haematology Unit with an enlarged inguinal lymph node which was diagnostic of a blastic plasmacytoid dendritic cell neoplasm. The staging revealed disseminated disease (skin, visceral, lymph nodes, and bone marrow). He received chemotherapy with an acute myeloid leukaemia-like regime. Afterwards, he underwent allogeneic haematopoietic stem cell transplantation, though it was not successful, showing a relapse 14 months later with hepatic and central nervous system dissemination. Intrathecal chemotherapy was administered, and venetoclax (anti-bcl2 agent) was started in an off-label indication based on most recent literature. The disease halted its course for 3 months. In the end, the patient’s disease progressed and so he succumbed due to infectious complications.

**Conclusions:**

Venetoclax monotherapy seems not enough to control the disease progression under CNS involvement and other treatments should be investigated.

## Background

Blastic plasmacytoid dendritic cell neoplasm (BPDCN) is an aggressive haematological malignancy which frequently presents with skin, bone marrow (BM), lymph node, and splenic infiltration at a median age of 67 years. Central nervous system (CNS) involvement and circulating leukaemia cells might be present (10%) [1]. There is no standard therapy, and patients frequently receive acute myeloid leukaemia (AML)-like regimens or are enrolled in clinical trials [2]. For patients who respond to chemotherapy, autologous or preferably allogeneic haematopoietic stem cell transplant (allo-HSCT) may prolong survival [3]. However, the median survival is approximately 1 year or even shorter for patients with disseminated disease. Age >60 years, abnormal karyotype and TdT negativity are some prognostic factors which have recently been associated with a lower overall survival [4].

A gene expression analysis suggested that BPDCN was similar to AML on a molecular expression basis, and identified that the anti-apoptotic B-cell lymphoma 2 (*BCL2*) gene is highly expressed in BPDCN compared to normal plasmacytoid dendritic cells. *BCL2* is also expressed in AML, and recently it has been reported that some cases of AML are sensitive to the Bcl-2 inhibitor venetoclax [5].

We present the case of a patient diagnosed with BPDCN that relapsed after allo-HSCT; venetoclax was considered the best therapeutic option based on recently published data.

## Case presentation

A 54-year-old Caucasian male with a history non-ST elevation myocardial infarction in 2015 was diagnosed with blastic plasmacytoid dendritic cell neoplasm in July 2016 due to an inguinal tumor. At the time of diagnosis a positron emission tomography (PET/CT) scan had revealed subcentimeter mildly hypermetabolic adenopathies in various territories (multiple lymph nodes involvement, hepatic, and splenic infiltration). He also had small skin plaques in lower extremities. The ganglion biopsy had shown an infiltration of 56% of atypical lymphocytes and peripheral blastosis, with a paracortical infiltration by Tdt^+^ cells, without expression of B or T markers while CD123 and CD56 had resulted positive, and a Ki-67 index of 60%, with negativity for CD34, CD3, CD20, myeloperoxidase, lysozyme. BM aspiration resulted dry and immunophenotype showed an abnormal CD4^+^, CD45^weak^, CD56^+^, CD123^+^ and HLA-DR^+^ cells (dendritic cells). BPDCN is characterized by high levels of CD123 and weak expression of CD45, while also being positive for CD4, CD36, CD56, as well as absence of lineage-associated antigens; this is considered a unique phenotype virtually pathognomonic of BPDCN [6].

He received 2 cycles of hyper-CVAD without intrathecal therapy (cyclophosphamide 300 mg/m^2^ BID days 1–4, vincristine 2 mg days 4 and 11, doxorubicin 25 mg/m^2^ q24h days 4–5, dexamethasone 40 mg QD days 1–4 and 8–11, methotrexate 1 g/m^2^ day 1, and cytarabine 3 g/m^2^ BID days 2–3) from July 2016 to November 2016, after which persisted a blast count of 11% in the bone marrow (partial response –PR-). The main complication was pulmonary oedema due to ventricular dysfunction secondary acute myocardial infarction which required implantation of a drug-coated stent on the circumflex artery. He continued with an AML-like scheme with high doses of ara-C (3 g/m^2^ days 1, 3 and 5) in December 2016, achieving a complete response (CR) assessed by BM biopsy and PET/CT.

Therefore, given the high risk of recurrence and the availability of an identical human leucocyte antigen (HLA) sibling, on March 2017 he underwent a reduced-intensity allo-HSCT (due to the history of two myocardial infarctions), conditioned with fludarabine 52 mg/day and busulfan 66 mg q6h plus graft-vs-host disease (GvHD) prophylaxis with sirolimus and tacrolimus. The number of haematopoietic stem cells infused was 5.02 x10^6^ CD34/Kg. One month after transplant, April 2017, a BM biopsy was performed showing CR. He developed mild skin chronic GvHD, which did not avoid immunosuppressant suspension until 6 months after allo-HSCT.

**Fig. 1:**
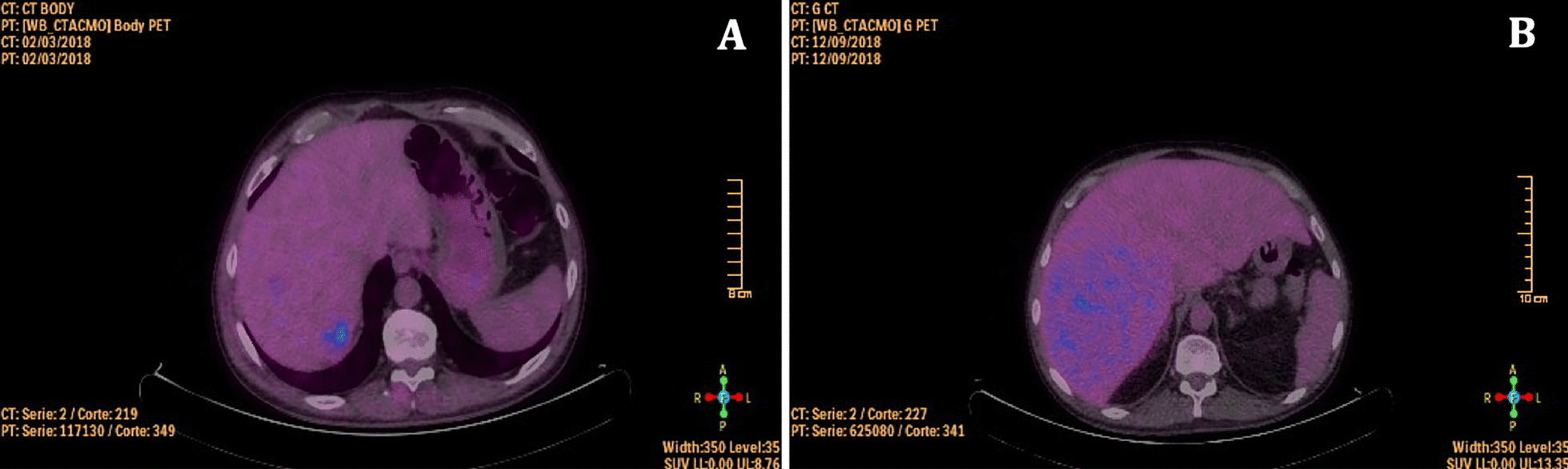
**A** PET/CT scan with non-specific hepatic lesions (March 2018). **B** PET/CT scan showing progression and liver enlargement under venetoclax treatment (September 2018)

**Fig. 2:**
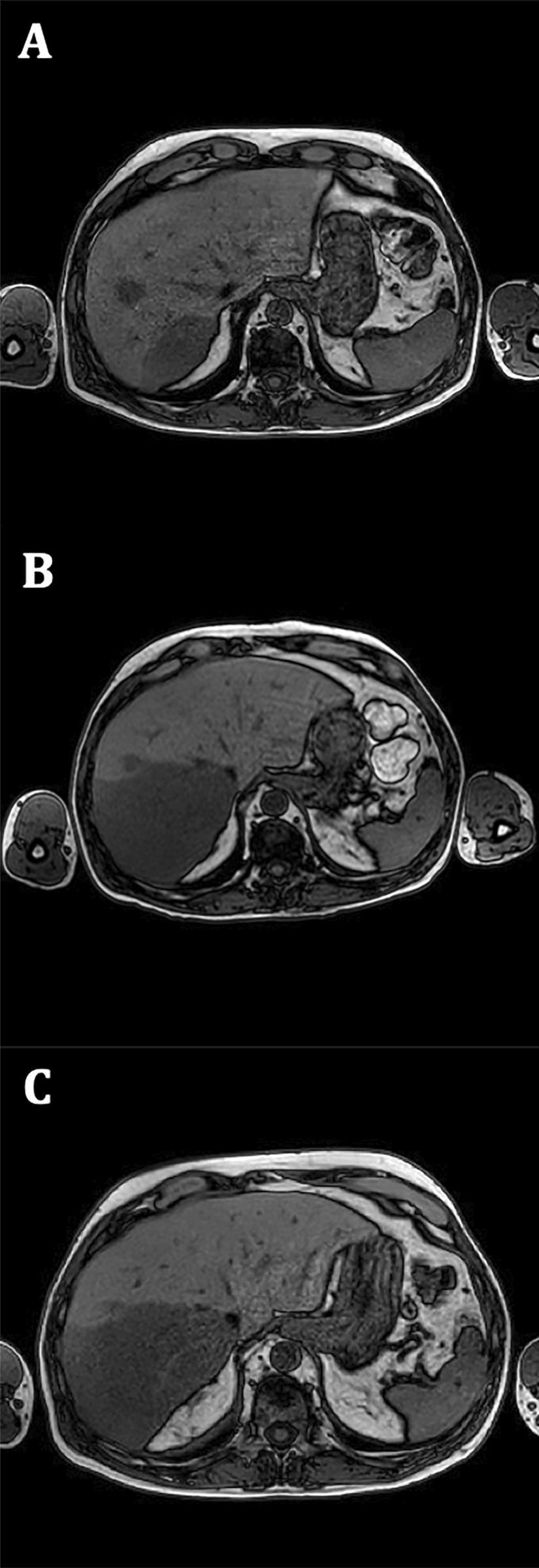
**A** Initial MRI showing liver spread of the disease (March 2018). Hepatic lesions were identified in segments VII and VIII of the liver. **B** Progression of the hepatic lesions. **C** Stability of the hepatic lesions under venetoclax treatment (August 2018)

**Fig. 3:**
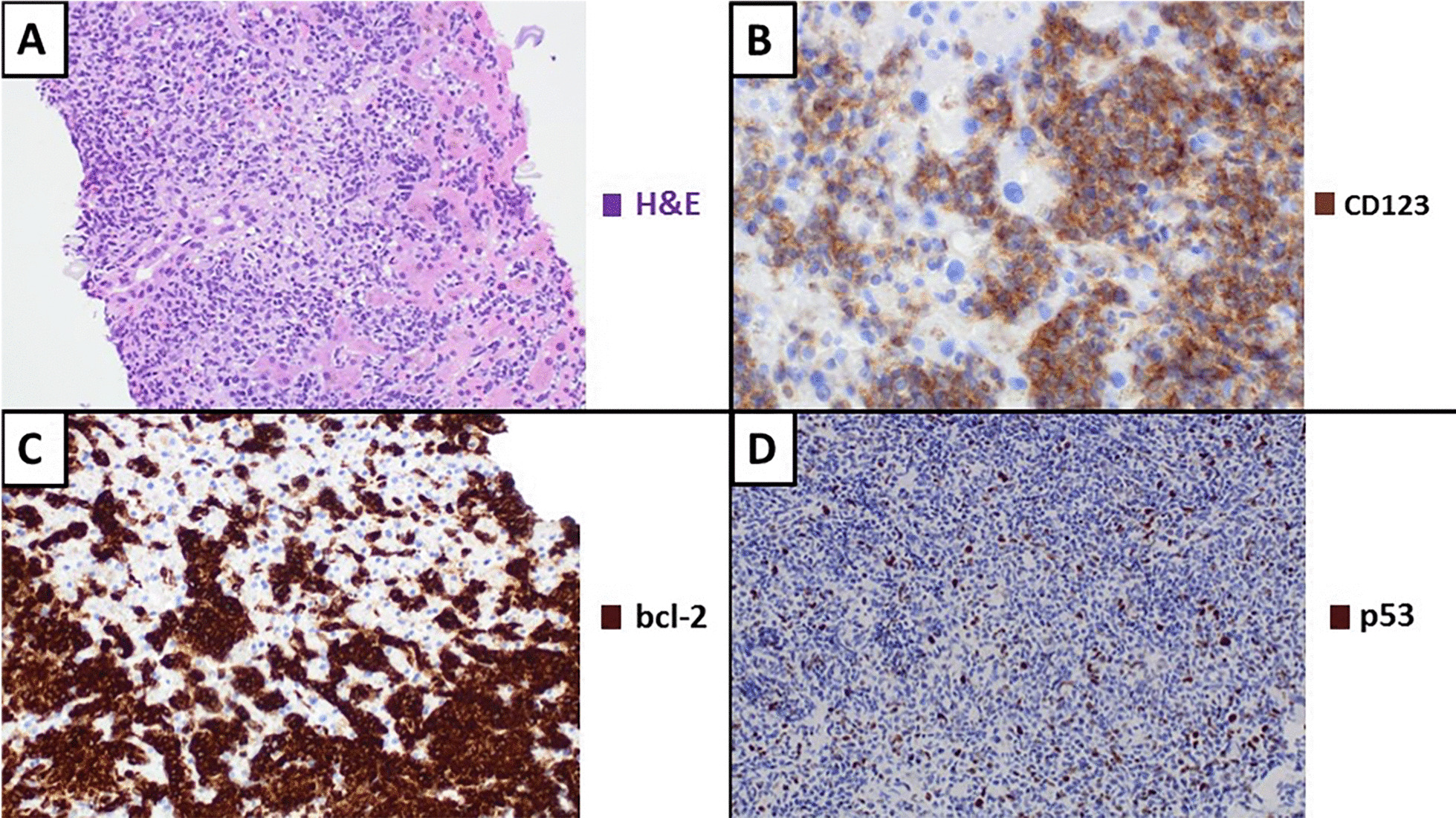
Histological sections of the liver biopsy showed a nodular infiltrate of blast cells, with ample cytoplasm, irregular nuclei and small nucleoli. There were occasional small lymphocytes intermingled with neoplastic cells. Tumour cells were diffusely positive for TdT, CD43 and CD56, as well as CD123. Only heterogeneous expression of CD4 was observed. **A** Nodular infiltration of the liver by blast cells. **B** Neoplastic cells show diffuse immunoreactivity for CD123. **C** Positive immunoreactivity for bcl-2. **D** Occasional intense positivity for p53 (less than 3%)

Nevertheless, 1 year later, March 2018, on a control PET/CT nonspecific hepatic lesions were evidenced (Fig. 1A), later confirmed by magnetic resonance imaging -MRI- (Fig. 2A) and tru-cut biopsy (Fig. 3); he then received intermediate dose ara-C (1.5 g/m^2^ days 1–5) and first infusion of donor lymphocytes on May 2018. One month after treatment start, a PET/CT and hepatic MRI (Fig. 2B) showed progression of one of the hepatic lesions and later on that same week he consulted with a short history of oppressive headache, photophobia, and tinnitus, without additional neurological symptoms. He also referred blurred vision lasting a few minutes and an episode of amaurosis fugax. The headache worsened with decubitus and improved with sitting. Analgesic treatment was optimised without improvement, so he consulted again the day after.

With suspicion of CNS dissemination, a cerebral CT scan was performed, which showed signs of leptomeningeal spread. A lumbar puncture was executed with a high-pressure fluid outlet, so the procedure was suspended due to the risk of intracranial hypertension.

**Fig. 4:**
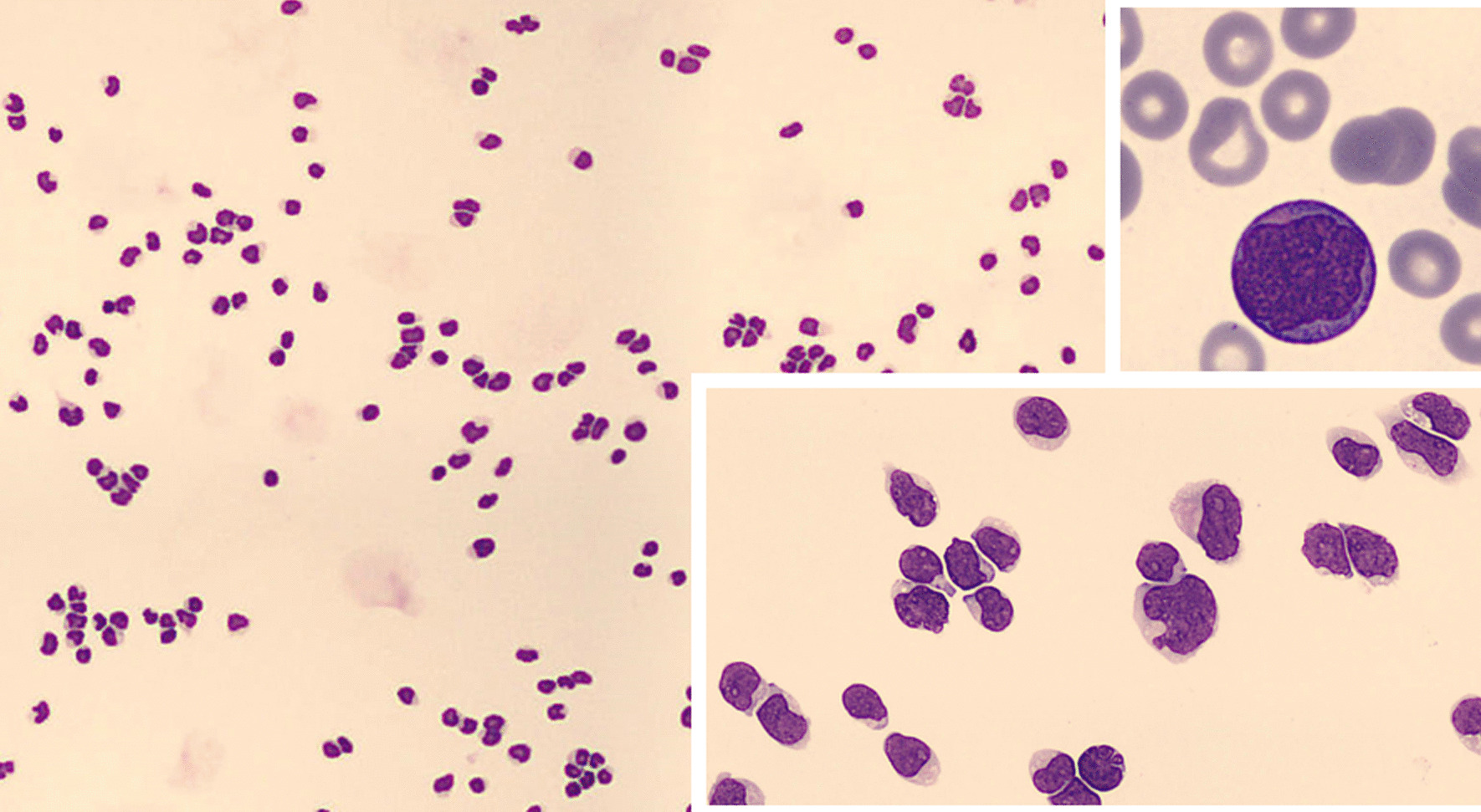
Cerebrospinal fluid smear (May-Grünwald-Giemsa staining) showing massive blast cell infiltration of the central nervous system. Lower right quadrant detail: zoom-in of blasts morphology in CNS. Upper right quadrant detail: blast morphology in peripheral blood. Blast cells are of large size and show a monocytic appearance, with irregular nuclei shape and some of them with nucleoli

Following the presumptive diagnosis of intracranial hypertension due to CNS dissemination of his leukaemia, dexamethasone (8 mg TID) was started and a new lumbar puncture was performed three days later administering triple intrathecal chemotherapy (methotrexate 12 mg, ara-C 30 mg, hydrocortisone 20 mg) thrice in July 2018. In cerebrospinal fluid (CSF), 1025 atypical cells were shown, compatible with dissemination of dendritic cell leukaemia (Fig. 4).

A week after, the 2nd dose was administered and a total of 165 cells were obtained. Three days after the 3rd dose was given obtaining 15 cells. No more CSF analysis was performed. All in all, 6 doses were given.

After extensive review of literature and based on other cases previously reported [5, 7–10], taking into account that malignant cells had a high expression of bcl-2 (Fig. 3C), venetoclax off-label was begun in an ascending scheme. The patient agreed to this off-label use. He started at a dose of 50 mg QD (July 2018) and gradually increased it up to 800 mg QD. After two months under treatment, on August 2018 an hepatic MRI was performed showing stability (Fig. 2C). However, on September 2018 a routine blood test suggested progression as liver enzymes and lactate dehydrogenase (LDH) increased while platelet levels went down. It was followed by a new BM aspirate and PET/CT, which confirmed progression under venetoclax treatment (Fig. 1B).

A palliative strategy was adopted, stopping venetoclax and starting hydroxyurea, azacytidine and dexamethasone. The patient eventually died one month and a half after stopping the treatment with venetoclax, on October 2018.

A post-mortem next generation sequencing (NGS) study was performed on the liver biopsy using the TruSight®Tumor 15 (Illumina) kit, which analyses the following 15 genes: *AKT1*, *BRAF*, *EGFR*, *ERBB2*, *FOXL2*, *GNA11*, *GNAQ*, *KIT*, *KRAS*, *MET*, *NRAS*, *PDGFRA*, *PIK3CA*, *RET*, and *TP53*. The bioinformatics analysis was performed through the software Variant Studio, focusing on these regions: *KRAS* (exons 2, 3, and 4), *NRAS* (exons 2, 3, and 4), *BRAF* (exon 15), *EGFR* (exons 18, 19, 20, and 21), *PIK3CA* (exons 10 and 21). None of them was mutated. However, a mutation in the exon 7 of *TP53* (p.Gly245Ser, g. 7577548, c.733G>A) was found in 57,4% of the samples.

The tru-cut biopsy had already showed intense expression of p53 in few cells that should be related to that mutation found in the NGS analysis, presumably correlating to a malfunctioning p53 protein, and thus leading to a poorer prognosis.

## Discussion and conclusions

Currently, the pathogenesis underlying the transformation of haematopoietic progenitors to BPDCN remains unclear. Using targeted sequencing techniques, some genes (e.g. *ASXL1*, *TP53*, *TET2*) have been found to be mutated in BPDCN as well as in other myeloid malignancies [7]. However, none was specific of BPDCN.

Venetoclax is a bcl-2 inhibitor currently authorised to treat chronic lymphocytic leukaemia (CLL). BPDCN also shows *BCL2* gene overexpression and *TP53* mutation [7]. Following this reasoning, it has been tested on BPDCN with favourable outcomes [5, 7, 8]. Montero *et al*. first described promising results with venetoclax both in vitro and in vivo (through xenografts) [7]. They also proved in mice an overall survival of 21 days.

At the time of giving venetoclax to our patient, there were 6 cases reported: one by Agha *et al*. [10] two also by Montero *et al*. [7] two by DiNardo *et al*. [5] and one by Grushchak *et al*. [8]. The first one responded very fast and maintained complete response for at least 9 months reported [10]. The two reported by Montero *et al*. had relapsed disease after CD123-targeting therapy and achieved a partial response at 4 weeks of venetoclax treatment: one of them progressed after 12 weeks and the other one died from intracranial haemorrhage probably secondary to severe venetoclax-induced thrombocytopaenia; the two included in DiNardo’s trial had received more than 2 prior lines of therapy and achieved cutaneous response with venetoclax; the remaining one relapsed after CHOP and auto-HSCT, after which received venetoclax achieving a CR during 10 months. Only in one of them a PET/CT response and >50% of blast reduction in bone marrow were reported.

When comparing these patients to ours, the median age was older (73,4), neither of them had CNS involvement nor were treated with allo-HSCT. Furthermore, in all the 5 cases specific techniques were used to predict bcl-2 inhibition response. Although we could not perform any of these techniques at the relapse, we decided to try it out in our patient, as the malignant cells overexpressed bcl-2 in the previous hepatic biopsy. The disease halted its course for two months. After that, it was reactivated reactivated. For that reason, venetoclax therapy was stopped.

Even though the patient had CNS disease, during venetoclax treatment period no neuroimaging techniques were performed. However, he did not show neurologic symptoms. Albeit venetoclax’s role in CNS has not been specifically studied, mainly because CLL rarely involves CNS, and there are no tests available in our centre to measure its concentration in CSF, there is evidence that suggests it can get to the CNS, though this has only been proved in mice [9].

Although disease progression stopped for two months, better results have been achieved in the previously described case reports. This may be due to the addition of a hypomethylating agent in some of them or due to the clinical features of the patients. We did not add a hypomethylating agent because at that time there was little evidence of its efficacy in combination therapy in BPDCN. Thus, the fact that neither of them presented CNS disease suggests a less aggressive subset of BPDCN. Moreover, as they were not treated with allo-HSCT, the disease might be more chemo-sensitive, considering they received less intense chemotherapy regimens. In fact, the patient who showed a CR of 10 months had only been treated with CHOP and auto-HSCT, whereas our patient received hyper-CVAD, AML-like scheme and reduced intensity conditioning regime for allo-HSCT plus intrathecal therapy. There is also the possibility that our patient’s disease was not fully sensitive to venetoclax, as we did not perform a bcl-2 overexpression test at relapse.

At the time of writing this paper, more results of venetoclax use are available. In another case report by Beziat *et al.*, the patient did not exhibit a very good response, as some cutaneous lesions remained stable while others responded [11]. Then, two other papers in combination therapy with hypometilating agents report pretty good results, with rapid and durable responses [12, 13]. A summary of cases in which venetoclax was used, including ours, is available in Table 1.

**Table 1.** Summary of cases available in the literature that used venetoclax in BPDCN

Study	*N*	Median age	Median previous lines	Added treatments	Type of response	Max. duration of response	Follow-up
Grushchak *et al*. Medicine (2017)[8]	1	65	2	No	CR	>10 months	10 months
Montero *et al*. Cancer Discov. (2017)[7]	2	76,5	3	No	PR	4 weeks	Until death
DiNardo *et al*. Am J Hematol. (2018)[5]	2	74,5	3	Yes, NR but probably HMA or LDAC	CR/PR	NR	NR
Agha *et al*. N Engl J Med. (2018)[10]	1	62	4	No	CR	>9 months	9 months
Beziat *et al*. Leuk Res. (2019)[11]	1	77	1	No	PR	1 month	NR
Piccini *et al*. Ann Hematol. (2020)[12]	1	64	2	Azacitidine	CR	>11 months	11 months
Samhouri *et al*. J Oncol Pharm Pract. (2020)[13]	1	79	1	Azacitidine	PR	>6 months	6 months
Albiol *et al*. J Med Case Rep. (2021)	1	54	4 plus intrathecal therapy	No	PR	3 months	Until death

*CR* complete response, *PR* partial response, *NR* not reported, *HMA* hypomethylating agent, *LDAC* low-dose ara-C

Taking everything into account, there is limited bibliography available involving blastic plasmacytoid dendritic cell neoplasms, and the treatment regimens are not based on the strongest level of evidence [1, 2]. Moreover, few cases are diagnosed each year and the survival rate of them is little to nothing.

The best therapeutic option for a newly diagnosed case of BPDCN is to be enrolled in a clinical trial or, if not available, to receive acute lymphoblastic leukaemia (ALL)-like regimens, AML-like regimens or a combination of both followed by an allo-HSCT if eligible. No statistical differences have been observed between using an ALL-like regime against an AML-like one [2]. However, a recent analysis by the MSKCC and two other US centres found statistically significant differences between ALL and AML-like regimens, suggesting a better overall survival (OS) if an ALL-like regime was used [4].

As of clinical trials, there are different approaches being tested right now. The most innovative seem to be those targeting CD123 (interleukin-3 receptor), as it is quite specific of blastic plasmacytoid dendritic cells. One of the most promising trials is a diphtheria toxin and IL-3 fusion protein -Tagraxofusp (Tagrax®)- also known as SL-401 [14, 15]. It is a CD-123 directed cytotoxin which has shown high response rates at the cost of little toxicity, so it enlightens the future of BPDCN management. It was approved by the Food and Drug Administration (FDA) in December 2018 and it is now recommended as a first line induction regime (evidence grade 2C) [16].

There are also antibodies against CD-123 (e.g. IMGN632, a CD123-Targeting Antibody-Drug Conjugate (ADC) with a deoxyribonucleic acid (DNA)-Alkylating Payload) and CAR-T cells [17], which are showing promising results but are still on preclinical phase due to safety profile issues [18].

Other promising agents include pralatrexate, an antifolate drug used in 8 patients with BPDCN achieving an overall response rate of 75% and 4 of them reaching complete response, and enasidenib, an IDH2 inhibitor which halted disease progression for 8 months in a patient with IDH2 mutation [4].

All in all, evidence suggests that venetoclax in combination with hypomethylating agents could be useful in some BPDCN patients, but in advanced stages it shows little efficacy, especially if it is used alone. Nevertheless, further studies are needed to demonstrate its real role. Currently, there is an on-going clinical trial (NCT03485547) which is trying to give more answers. Moreover, considering the infrequency of this disease it is unlikely that in the nearly following years we can get any strong evidence about it.

## Data Availability

Data sharing is not applicable to this article as no datasets were generated or analysed during the current study.

## Abbreviations

BPDCN: Blastic plasmacytoid dendritic cell neoplasm
BM: Bone marrow
CNS: Central nervous system
AML: Acute myeloid leukaemia
allo-HSCT: Allogeneic haematopoietic stem cell transplant
DNA: Deoxyribonucleic acid
PET/CT: Positron emission tomography/computerised tomography scan
PR: Partial response
CR: Complete response
FDA: Food and Drug Administration
GVHD: Magnetic resonance imaging (MRI)
HLA: Human leucocyte antigen
TID: Ter in die
CSF: Cerebrospinal fluid
QD: Quaque die
BID: Bis in die
NGS: Next Generation Sequencing
CLL: Chronic Lymphocytic Leukaemia
ALL: Acute lymphoblastic leukaemia
MSKCC: Memorial Sloan Kettering Cancer Center
OS: Overall survival
ADC: Antibody-Drug Conjugate
CAR-T cells: Chimeric Antigen Receptor T cells
